# The integrated treatment of eating disorders, posttraumatic stress disorder, and psychiatric comorbidity: a commentary on the evolution of principles and guidelines

**DOI:** 10.3389/fpsyt.2023.1149433

**Published:** 2023-05-12

**Authors:** Timothy D. Brewerton

**Affiliations:** Department of Psychiatry and Behavioral Sciences, Medical University of South Carolina, Charleston, SC, United States

**Keywords:** eating disorders, trauma, PTSD, comorbidity, treatment, guidelines

## Abstract

Psychiatric comorbidity is the norm in the assessment and treatment of eating disorders (EDs), and traumatic events and lifetime PTSD are often major drivers of these challenging complexities. Given that trauma, PTSD, and psychiatric comorbidity significantly influence ED outcomes, it is imperative that these problems be appropriately addressed in ED practice guidelines. The presence of associated psychiatric comorbidity is noted in some but not all sets of existing guidelines, but they mostly do little to address the problem other than referring to independent guidelines for other disorders. This disconnect perpetuates a “silo effect,” in which each set of guidelines do not address the complexity of the other comorbidities. Although there are several published practice guidelines for the treatment of EDs, and likewise, there are several published practice guidelines for the treatment of PTSD, none of them specifically address ED + PTSD. The result is a lack of integration between ED and PTSD treatment providers, which often leads to fragmented, incomplete, uncoordinated and ineffective care of severely ill patients with ED + PTSD. This situation can inadvertently promote chronicity and multimorbidity and may be particularly relevant for patients treated in higher levels of care, where prevalence rates of concurrent PTSD reach as high as 50% with many more having subthreshold PTSD. Although there has been some progress in the recognition and treatment of ED + PTSD, recommendations for treating this common comorbidity remain undeveloped, particularly when there are other co-occurring psychiatric disorders, such as mood, anxiety, dissociative, substance use, impulse control, obsessive–compulsive, attention-deficit hyperactivity, and personality disorders, all of which may also be trauma-related. In this commentary, guidelines for assessing and treating patients with ED + PTSD and related comorbidity are critically reviewed. An integrated set of principles used in treatment planning of PTSD and trauma-related disorders is recommended in the context of intensive ED therapy. These principles and strategies are borrowed from several relevant evidence-based approaches. Evidence suggests that continuing with traditional single-disorder focused, sequential treatment models that do not prioritize integrated, trauma-focused treatment approaches are short-sighted and often inadvertently perpetuate this dangerous multimorbidity. Future ED practice guidelines would do well to address concurrent illness in more depth.

## Trauma, PTSD, and eating disorders

Scientific evidence for the association between trauma, PTSD and other trauma-related disorders in the predisposition, precipitation and perpetuation of eating disorders (EDs), has been established in a variety of samples, including treatment and non-treatment seeking individuals of various ages, genders and sexual orientations (1–6). The pooled lifetime prevalence rates of PTSD in EDs (ED + PTSD) in the highest quality studies average 25% with higher rates of 37–45% in bulimia nervosa (BN) and 21–26% in binge eating disorder (BED) (7–9). Lower prevalence rates are reported in association with AN, particularly the restricting type (10–14%). Nevertheless, higher rates of PTSD are reported in all ED patients admitted to residential care, especially those with BN, anorexia nervosa binge-purge type (AN-BP), and other specified feeding and eating disorders (OSFED) (2, 10, 11). Conversely, higher rates of EDs are also seen in patients with PTSD compared to the general population (8, 12).

It is common for ED + PTSD patients to report histories of multiple traumas and/or trauma types (1, 2, 10, 13). High trauma “doses” have been associated with ED severity and comorbidities (2, 13–16), and trauma histories and PTSD symptoms have been reported to predict more complicated courses of illness, higher dropout rates, and worse outcomes following treatment (11, 17–26). Evidence also suggests that individuals with ED + PTSD may be significantly more impulsive, prone to revictimization, and the subsequent perpetuation of PTSD (27–30), which apart from EDs tends to be a chronic disorder (31–33). Taken together, the development and adoption of integrated treatment approaches for ED + PTSD is warranted.

## Existing ED guidelines lack a trauma-PTSD focus

Existing guidelines for the treatment of EDs do not adequately address the problem of psychiatric comorbidity, which is more common than not in this group of disorders. Although admittedly this is a complicated topic, previously published ED guidelines often mention the entire issue of psychiatric comorbidity in a rather cursory manner and, if mentioned at all, do not address the depth of discussion or nuance that is often necessary to address ED + PTSD. In the excellent 2017 review of 8 existing guidelines for AN by Hilbert and colleagues, only 3 of these 8 manuscripts mentioned psychiatric comorbidities, while 5 of 9 guidelines for BN noted comorbidities, usually as “*special issues*” (34).

The issue of trauma and specifically of PTSD in the assessment of EDs was noted in the Australian and New Zealand guideline. These experts noted that psychiatric comorbidity occurs as high as 55% in community adolescent samples and up to 96% in adult samples, and they state, “*Comorbidity in people with anorexia nervosa is common and therefore assessment for such should be routine*” (35). They also make the recommendation to “*be prepared to treat comorbidity to improve quality of life*,” although no specifics as to how to do this are offered other than using CBT-E, which does not directly address trauma or PTSD, and considering adjunctive pharmacotherapy (35).

The updated German guidelines for the treatment of AN specifically noted, “*many patients are affected by comorbid psychological diseases*,” and that comorbid conditions, such as PTSD, “*might require changes in treatment planning and prioritization of therapy goals*,” although no specifics were provided (36).

More recently the American Psychiatric Association published an updated practice guideline for the treatment of patients with eating disorders, which notes that “*eating disorders frequently co-occur with other psychiatric disorders*” (37, 38). The guideline states that “*all patients with a possible eating disorder should be asked about a history of trauma … and assessed for symptoms related to PTSD*.” Although “*data are…limited on individuals with eating disorders and…co-occurring psychiatric disorders*,” the guideline recommends that psychotherapy in adults with AN “*address comorbid psychopathology, psychological conflicts, adaptive benefits of symptoms, and family or cultural factors that reinforce or maintain eating disorder behaviors*,” suggestions which are certainly applicable to the treatment of ED + PTSD in patients with any type of ED.

Notably, none of the guidelines for adult patients with EDs that were reviewed for this commentary specifically addressed treatment of ED + PTSD *per se*. However, in the newly published Australian guideline for the treatment of ED patients at higher weights, much more attention to trauma, PTSD, and trauma-informed care is paid (39). This is relevant given that ED patients with PTSD treated in higher levels of care have been reported to have significantly higher BMIs (2, 10, 40). Importantly, Ralph and colleagues offer lived experience perspectives, and specifically discuss trauma-informed care within an ED context for patients in larger bodies. They also acknowledge how ED treatment itself may be traumatizing and how PTSD and other comorbidities occur “*frequently*” (39).

Three published guidelines were found for the treatment of children and adolescents with EDs (41–43). The focus on psychiatric comorbidity was most prominent in the guideline from the American Academy of Child and Adolescent Psychiatry, which states “*further complicating the diagnosis of AN is the potential presence of other significant psychiatric comorbid conditions*” (43). Abuse is noted to be a potential risk factor for BN, and PTSD an associated comorbid disorder in BN and BED, but no specific treatment guidelines for ED + PTSD are provided other than to recommend that “*guidelines for the specific condition should be followed*” and “*The use of medications, including complementary and alternative medications, should be reserved for comorbid conditions and refractory cases*.” The Canadian guideline for children and adolescents with EDs only mentions trauma and PTSD in reference to ARFID (41). However, it notes in the very last line of the guidelines the following statement: “*Research efforts should be devoted to developing treatments for severe eating disorders with complex comorbidity*.”

## Existing PTSD guidelines lack an eating disorder focus

Conversely, upon reviewing available practice guidelines for the assessment and treatment of PTSD or complex PTSD, the treatment of EDs is never addressed (44–54). The only PTSD guidelines that specifically mention EDs as an associated comorbidity are those of the American Psychiatric Association (55). Several guidelines specifically note that the presence of comorbid disorders should not prevent patients from receiving trauma-focused treatments (51, 52, 54, 56). However, in my experience trauma specialists are often unprepared to deal with PTSD patients with active ED symptoms. Unfortunately, it has been a common but unfortunate occurrence for ED + PTSD patients to be refused treatment in either an ED specialty program or trauma specialty program, or if they are admitted, only the so-called “primary” disorder (for insurance purposes) is treated. The treatment of one disorder to the exclusion of the other is often inadequate in that it all too often results in poor outcomes.

## Toward the development of treatment guidelines for ED + PTSD and related comorbidity

**Figure 1:**
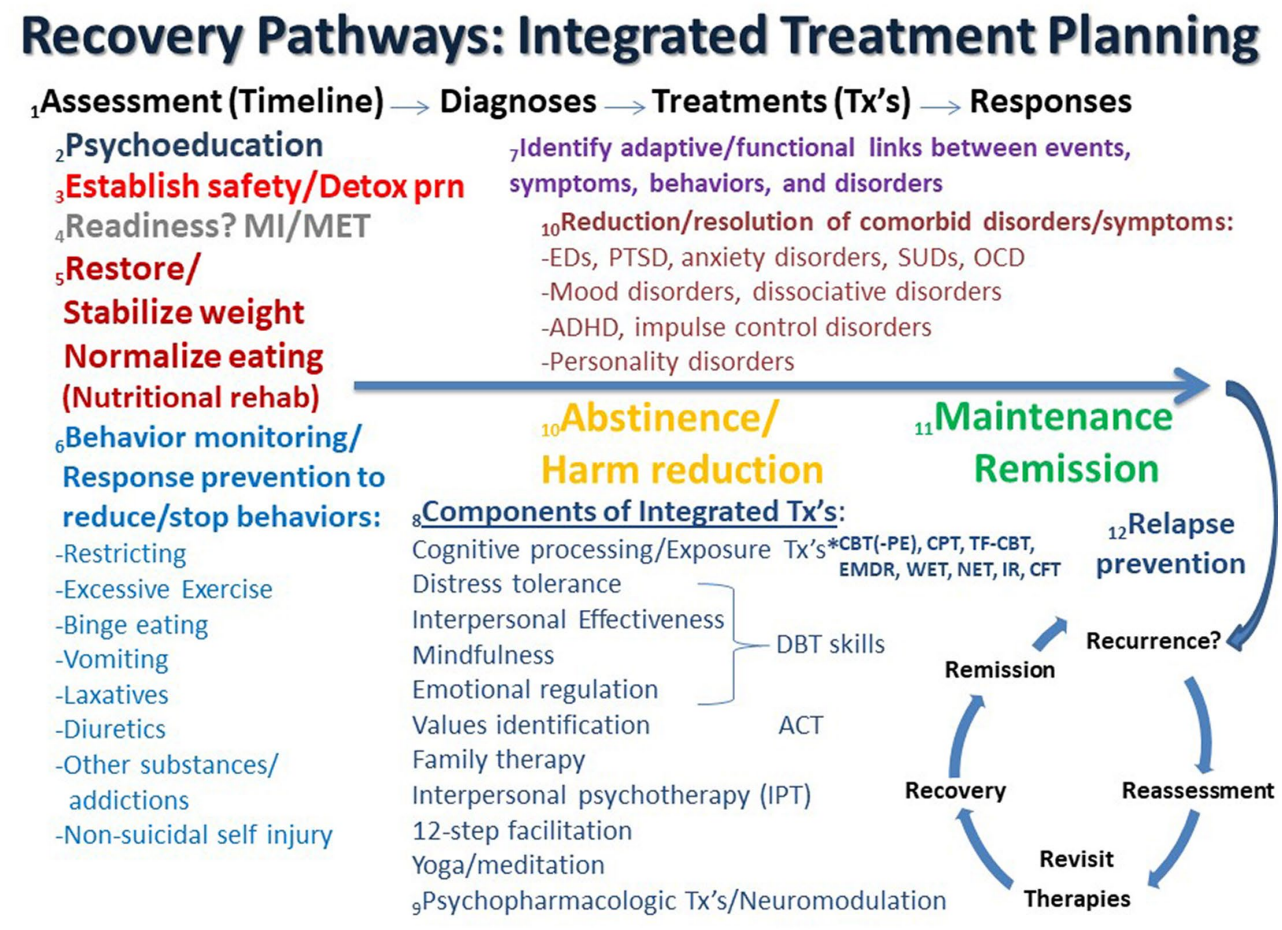
Recovery pathways that facilitate integrated treatment planning are discussed consecutively in the text as numbered. ACT, acceptance commitment therapy; ADHD, attention deficit hyperactivity disorder; CBT, cognitive behavioral therapy; CFT, compassion focused therapy; CPT, cognitive processing therapy; Detox, detoxification; DBT, dialectical behavior therapy; EMDR, eye movement desensitization reprocessing; IR, imaging rescripting; IPT, interpersonal psychotherapy; MI, motivational interviewing; MET, motivational enhancement therapy; NET, narrative exposure therapy; OCD, obsessive compulsive disorder; PTSD, posttraumatic stress disorder; PE, prolonged exposure; SUD, substance use disorder; TF-CBT, trauma-focused cognitive behavioral therapy; Tx’s, treatments; WET, written exposure therapy.

Recommendations regarding the treatment of ED + PTSD and related comorbidity have been previously outlined and discussed (1, 57, 58), and an updated, expanded conceptualization of these guidelines and principles is discussed here. Possible recovery pathways that may facilitate integrated treatment planning are shown in Figure 1. In the figure, the process starts on the left and proceeds in time to the right from Assessment (Timeline) to Diagnosis to Treatment (Tx) to Response as the patient ideally proceeds from highly symptomatic on the left to maintenance and/or remission on the far right, followed by relapse prevention and/or recurrence and reassessment.

**1. Assessment (Timeline) – Diagnosis:** Most guidelines recognize the importance of “*a comprehensive assessment of the individual and their circumstances*” (35). It has been previously noted that “*the most tried and true initial approach for any patient with or without complex symptomatology is to perform a complete psychiatric evaluation, which substantially reduces the chances of misdiagnosis*” (57). This remains as important as ever, especially in an era in which time-limited evaluations are the norm in most treatment settings. Therefore, assessment is often an ongoing process that unfolds over several sessions. As part of this complete medical and psychiatric evaluation, the clinician and patient collaboratively develop a timeline that depicts the chronology of significant life events, traumas, symptom/disorder onsets, remissions, and relapses in the individual’s lifetime. Such a timeline can form the basis of the patient’s life narrative, including history of traumatic events and important contexts, and allows the patient and clinician to recognize and discuss predisposing, precipitating, perpetuating, and palliative factors in the patient’s life. Such an approach is compatible with the emerging paradigm of a life course approach as well as the concept of personalized medicine to address multimorbidity (59–63). It is important to note that the development of the patient’s historical timeline may occur over an extended period of time as therapy progresses and evolves (see #1 in Figure 1). Many ED patients do not feel comfortable revealing traumatic events during the initial assessment but need time to develop a trusting alliance with the therapist before such history is disclosed. The formation and maintenance of a therapeutic alliance remains a cornerstone of future work and progress (64, 65). Therapists need to convey a sense of safety, trust, honesty, straightforwardness, compassion, knowledge, and nonjudgmental positive regard, which are essential elements for any successful treatment course. Countertransference also needs to be closely monitored and contained (66–68). Cognitive processing therapy (CPT) emphasizes the importance of therapists avoiding avoidance of addressing traumatic material (69), as avoidance is highly characteristic of both EDs and PTSD (6, 11, 70–75). It has been previously noted that it is not uncommon for patients with ED + PTSD to not even realize that they have been victimized until the definitions of trauma and abuse types are explained to them (57). This is particularly relevant for patients with child maltreatment and complex PTSD. Only later after a substantial degree of cognitive development has occurred do some individuals realize the extent and meaning of their traumas.

**2. Psychoeducation:** As a comprehensive list of problems and conditions is being determined, the clinician moves forward educating the patient and supportive family about all diagnosed disorders, both current and lifetime. Psychoeducation is the cornerstone of all modern therapies, especially cognitive-behavioral and trauma-focused approaches (see #2 in Figure 1). Psychiatric disorders often vacillate in and out of remission, and previous disorders may serve as important predisposing factors for subsequent symptoms and disorders. Notably, comorbidity is the rule rather than the exception in PTSD and EDs (57, 76–78), and comorbid disorders are generally not contraindications to trauma-focused treatments (79, 80). Such treatments can be safely and effectively used in patients with a range of psychiatric disorders and are often associated with concurrent decreases in PTSD and the comorbid problem(s) (80–83).

**3. Safety and stability:** The most important determination and goal early on in treatment is to initially assess and address the greatest danger or risk to life, e.g., suicidality, self-harming behaviors, starvation, fluid/electrolyte disturbances, cardiac arrhythmia, etc. (see #3 in Figure 1). Attaining relative safety and stability is the foundation of stage 1 treatment of PTSD with complex presentations (complex PTSD), as well as dissociative disorders, which are highly linked to developmental traumas (46, 84–87). Given its pervasive association with problems in self-regulation, ED + PTSD with related comorbidities may be conceptualized as forms of complex PTSD (1, 88–90). Emerging behaviors that derail treatment progress, or therapy interfering behaviors (91–93), may need to be addressed as the therapist assists the patient to identify avoidance and work on stuck-points, cognitive distortions, or maladaptive beliefs (69). However, some trauma experts question the need for a phasic approach, arguing that there is a lack of research to support several contentions, including that: (1) a phase-based approach is necessary to attain positive outcomes, (2) trauma-focused treatments have unacceptable risks, and (3) response to trauma-focused treatments are improved when preceded by a stabilization phase (94). On the other hand, ISTSS guidelines indicate sufficient evidence and clinical rationale to support the phase-oriented approach with an initial period of stabilization and establishment of safety (46, 84, 95). Suicidality and other immediately life-threatening medical conditions are effectively the major primary contraindications against initiating trauma-focused treatment (79). When a substance use disorder (SUD) complicates the clinical picture, a detox strategy that is appropriate for the particular substance is an important early treatment component that is subsumed under the goal of establishing safety and stability.

**4. Readiness for change:** As assessment and treatment move forward, it is incumbent on therapists to identify readiness for change and willingness to engage in both PTSD and ED recovery, which are intimately interwoven (96–100). Motivational interviewing (MI) and motivation enhancement therapy (MET) approaches may help to identify the most problematic condition per the patient’s perspective and can be important prequels and/or adjuncts to other subsequent evidence-based therapies for EDs, PTSD and related comorbidities (101–108) (see #4 in Figure 1). However, it is notable that readiness for trauma-focused treatment is not always accurately assessed by therapists, which can lead to inadvertent collusion with avoidance (101).

**5. Nutritional rehabilitation:** Early on in treatment it is essential to establish that the patient has begun the process of nutritional rehabilitation, is more adherent to this plan than not (progress not perfection), is medically stable, and can begin to process information emotionally and cognitively (see #5 in Figure 1). New learning is dependent upon adequate food intake and subsequent protein and neurotransmitter synthesis (109–111). Ideally, effective psychotherapy requires grossly intact brain function, the ability to attend to the process at hand, and the ability and motivation to learn new information (and unlearn maladaptive strategies). Starved, intoxicated and/or severely dysregulated brains are unable to learn well and therefore are less likely to benefit from psychotherapy. Nevertheless, trauma-focused treatment should not be delayed until full weight restoration or remission of ED behaviors is achieved as long as the patient is safe and willing to engage in trauma processing. Evidence has been accumulating that the long-term resolution of ED symptoms may in fact hinge on trauma-focused treatment (4, 77, 112, 113).

**6. Behavior monitoring and response prevention:** Just as psychoeducation is an integral early component of cognitive behavioral therapies, so too is behavior monitoring and response prevention of problematic behaviors. Patients are asked to record instances and frequencies of targeted behaviors, including food intake and episodes of binge eating, purging, excessive exercise, substance use, and non-suicidal self-injury. This prescription is not only a part of ongoing assessment but is an effective treatment intervention in itself (see #6 in Figure 1). As treatment continues, the therapist periodically confirms that ED behaviors are being sufficiently addressed in an ongoing manner. Monitoring of ED and PTSD symptoms using validated measures, e.g., the Eating Disorder Examination Questionnaire (EDEQ) (114) and the PTSD Symptom Checklist for DSM-5 (PCL-5) (115), respectively, are essential in assessing progress for the ED + PTSD patient, while an array of other assessments are available for other comorbidities. While full remission may remain elusive, it is helpful to frame various degrees of symptom improvement as evidence of progress and success. Combating perfectionistic, “all or nothing” thinking is often an ongoing process in the long-term treatment of ED + PTSD patients, and teaching and promoting the concept and goal of harm reduction is a useful endeavor that has application to multiple symptoms and disorders on the comorbidity spectrum (116–118).

**7. Adaptive function:** An important part of the therapeutic process is to collaboratively identify the relationships between life events, symptoms of PTSD, eating and other disorders, which may serve as adaptive functions, such as to facilitate avoidance and numbing, decrease hyperarousal, regulate trauma-related states (“self-medication hypothesis”) (119) or solve some perceived problem(s) (15). Certain ED behaviors may also be traumatic reenactments (120). “Building the bridge” between trauma-related/PTSD symptoms and ED behaviors/symptoms serves to foster new connections and meanings and paves the way toward sustained recovery (see #7 in Figure 1). Continued work on the patient’s timeline can facilitate ascertaining these connections. Establishing the interconnectedness of and bridging between symptoms is supported by recent network analyses of ED and PTSD symptoms (97, 99, 100).

**8. Components of integrated treatment: psychotherapies:** It is argued that an integrated trauma-focused treatment plan using a rational mixture of evidence-based techniques and tools is indicated. An overview of the psychotherapeutic components that may be considered in integrated treatment planning is shown in Figure 1 (see #8). The field has evolved beyond the “one size fits all” approach, which is woefully inadequate for the ED + PTSD patient. Generally, some form of trauma-focused treatment is indicated for PTSD, and this should be no different when there is ED comorbidity. The most researched of trauma-focuses approaches in this population is cognitive processing therapy (CPT), which arguably is very well-suited for ED + PTSD patients (29, 112, 113, 121–123). However, other trauma-focused therapies can be applied to ED + PTSD patients, including prolonged exposure (PE), eye movement desensitization reprocessing (EMDR), imaging rescripting (IR), written exposure therapy (WET), narrative exposure therapy (NET), compassion-focused therapy (CFT), and trauma-focused CBT (TFCBT) in children and adolescents (49, 71, 121, 124–142). Other adjunctive approaches, or elements of these approaches, can also be useful, including acceptance and commitment therapy (ACT), which identifies values and addresses experiential avoidance (143–146), interpersonal psychotherapy (IPT), which has been reported to be effective in EDs, PTSD, and major depression (145, 147–152), and 12-step facilitation, a manual based, therapist driven treatment based on 12-step principles found to be effective for alcohol use and stimulant use disorders (153–155). The unified treatment model has also been recommended for ED + PTSD (156). Self-administered emotional freedom techniques (EFT) have been successfully employed for PTSD, but have not been systematically explored in EDs (157). Family therapy with non-offending parents and/or guardians is an essential ingredient for children and adolescents with ED and PTSD, and it should be considered for all patients of any age (158, 159). Involving family members in assessment and treatment is often extremely helpful and can contribute to significant therapeutic advances. When patients are not amenable to involving family members it is important for therapists to understand why.

A sufficient level of distress tolerance and emotional regulation is required so that psychotherapy can proceed, although pauses and delays are likely to occur in the course of addressing patients with complex psychopathology. The use of DBT skills as grounding techniques to enhance self-regulation, mindfulness, distress tolerance and interpersonal effectiveness can be essential, powerful adjuncts to trauma-focused therapies for severely ill comorbid ED + PTSD patients (160, 161). Other grounding or anxiety reduction techniques, such as focused breathing, yoga and other meditation practices, can be utilized as evidence-based treatment adjuncts (162–172). However, a certain amount of emotionality is to be expected and should not in itself be a reason for delay. A central tenet of evidence-based trauma-focused treatment is for the therapist to “avoid avoidance” and to not collude with the patient’s tendency to deflect (69). Disengagement coping has been found to predict revictimization, while engagement coping predicts better outcomes (173).

**9. Components of integrated treatment: psychopharmacology and neuromodulation:** Psychopharmacological interventions are also effective evidence-based treatments for non-comorbid EDs, PTSD and related comorbid disorders, e.g., mood and anxiety disorders, but they should always be adjunctive to psychotherapeutic interventions for patients with ED + PTSD and related comorbidities (174–179) (see #9 in Figure 1). The agents that have been found to be beneficial in ED and PTSD samples separately generally include antidepressants, especially fluoxetine and other serotonin reuptake inhibitors, atypical antipsychotics, especially olanzapine, and anticonvulsants, especially topiramate (35, 180–199). In addition, naltrexone can be highly effective in reducing alcohol intake as well as combating non-suicidal self-injury and binge eating (200–210). Other novel approaches to consider, especially when treatment-refractory major depression is a focus, may include the use of newer psychopharmacologic agents, e.g., 5-HT4 receptor antagonists (211), combining other previously unused evidence-based psychotherapies and psychopharmacologic approaches (142, 212), adjunctive application of neuromodulation tools, such as repetitive transcranial magnetic stimulation (rTMS) (213, 214), deep TMS (215) or electroconvulsive therapy (ECT) (216), novel psychotropic-or psychedelic-assisted therapies, such as ketamine/esketamine, 3,4-methylenedioxymethamphetamine (MDMA), psilocybin, and ayahuasca (12, 28, 212, 217–231), as well as deep brain stimulation (DBS) (232–235), although many of these newer approaches continue to be preliminary, experimental and/or challenging to acquire.

**10. Resolution, abstinence, and harm reduction:** It is well known that improvement in one set of symptoms often results in the resolution of or improvement in related symptoms of other disorders, which exist in a network with each other (see #10 in Figure 1) (147, 236–238). For example, as ED symptoms abate with treatment so also can symptoms of disorders of mood, anxiety, personality, etc. (11, 112, 122, 239, 240). Similarly, abstinence from or a reduction in SUD behaviors often accompanies similar reductions in mood, anxiety and other comorbid symptoms (241, 242). Likewise, recovery from PTSD often results in improvements in a host of related psychological symptoms (243–245).

**11. Maintenance, remission, and relapse prevention:** As improvements continue and degrees of remission are achieved, patients may move into the maintenance phase of treatment (see #11 in Figure 1). It is during this phase that relapse prevention strategies can be successfully employed. Specific forms of CBT and pharmacotherapy focused on relapse prevention have been found to significantly reduce relapse for eating, substance use, mood and anxiety disorders (246–255).

**12. Refractoriness and relapse:** In the face of refractoriness to treatment, inadequate response, and/or relapse, which is common in EDs and related comorbidities (256–258), it is helpful for the patient and therapist to re-evaluate the treatment plan, identify triggers, stuck points, and potential weak points, and to revisit therapeutic options (see #12 in Figure 1). Experienced clinicians are familiar with the “whack-a-mole” phenomenon in which one or more problems resolve as others emerge. When the therapeutic skills of the therapist do not match the needs of the patient, then it is sometimes appropriate and in the best interests of the patient to discuss switching gears and referring to someone else with greater expertise, experience or skills. However, it is essential that hope continue to live eternal while the odds of attaining desired goals are evaluated. The lived experiences of patients with ED + PTSD, particularly those with severe and enduring eating disorders, should be carefully considered in light of what is and is not known about long-term outcome data (259–270). Despite the availability in some locations of euthanasia and physician-assisted suicide for so-called “terminal anorexia,” it is my and others’ opinion that this course of action not be considered a possible option (263, 271–273), while palliative care may be indicated and considered (274). There are so many potential therapeutic options and combinations of options that one is hard pressed to argue that all avenues have been exhausted in the face of ED + PTSD chronicity and refractoriness.

In conclusion, effective treatment of ED + PTSD and related comorbidity requires the therapist to acquire many needed skills and resources, many of which are outlined in Figure 1 and discussed in this commentary. Abraham Maslow once said, “*If all you have is a nail, then everything is a hammer*.” Applying only one therapeutic approach to every ED patient is insufficient, a conclusion that is hopefully obvious at this juncture. Available clinical research evidence suggests that continuing with traditional single-disorder focused, sequential treatment models with multiple providers that do not prioritize integrated, trauma-focused treatment approaches are short-sighted and often inadvertently perpetuate this dangerous multimorbidity. Future ED practice guidelines would do well to address concurrent illness in more depth. Taken together, these principles offer the clinician a map for better negotiating a successful journey of recovery for the ED + PTSD comorbid patient. Recently published outcome data indicate that integrated treatment approaches can result in significant improvement in ED, PTSD and related symptoms (11, 12, 112, 122, 127, 156).

## Author contributions

The author confirms being the sole contributor of this work and has approved it for publication.

## Conflict of interest

TB is the owner of Timothy D. Brewerton, MD, LLC.

## Publisher’s note

All claims expressed in this article are solely those of the authors and do not necessarily represent those of their affiliated organizations, or those of the publisher, the editors and the reviewers. Any product that may be evaluated in this article, or claim that may be made by its manufacturer, is not guaranteed or endorsed by the publisher.
